# Barriers and Facilitators to Home Computer and Internet Use Among Urban Novice Computer Users of Low Socioeconomic Position

**DOI:** 10.2196/jmir.9.4.e31

**Published:** 2007-10-22

**Authors:** Emily Z Kontos, Gary G Bennett, K Viswanath

**Affiliations:** ^1^Department of Society, Human Development and HealthHarvard School of Public HealthBoston, MAUSA; ^2^Dana-Farber Cancer InstituteBoston, MAUSA

**Keywords:** Digital divide, health information seeking, health disparities

## Abstract

**Background:**

Despite the increasing penetration of the Internet and amount of online health information, there are significant barriers that limit its widespread adoption as a source of health information. One is the “digital divide,” with people of higher socioeconomic position (SEP) demonstrating greater access and usage compared to those from lower SEP groups. However, as the access gap narrows over time and more people use the Internet, a shift in research needs to occur to explore how one might improve Internet use as well as website design for a range of audiences. This is particularly important in the case of novice users who may not have the technical skills, experience, or social connections that could help them search for health information using the Internet. The focus of our research is to investigate the challenges in the implementation of a project to improve health information seeking among low SEP groups. The goal of the project is not to promote health information seeking as much as to understand the barriers and facilitators to computer and Internet use, beyond access, among members of lower SEP groups in an urban setting.

**Objective:**

The purpose was to qualitatively describe participants’ self-identified barriers and facilitators to computer and Internet use during a 1-year pilot study as well as the challenges encountered by the research team in the delivery of the intervention.

**Methods:**

Between August and November 2005, 12 low-SEP urban individuals with no or limited computer and Internet experience were recruited through a snowball sampling. Each participant received a free computer system, broadband Internet access, monthly computer training courses, and technical support for 1 year as the intervention condition. Upon completion of the study, participants were offered the opportunity to complete an in-depth semistructured interview. Interviews were approximately 1 hour in length and were conducted by the project director. The interviews were held in the participants’ homes and were tape recorded for accuracy. Nine of the 12 study participants completed the semistructured interviews. Members of the research team conducted a qualitative analysis based on the transcripts from the nine interviews using the crystallization/immersion method.

**Results:**

Nine of the 12 participants completed the in-depth interview (75% overall response rate), with three men and six women agreeing to be interviewed. Major barriers to Internet use that were mentioned included time constraints and family conflict over computer usage. The monthly training classes and technical assistance components of the intervention surfaced as the most important facilitators to computer and Internet use. The concept of received social support from other study members, such as assistance with computer-related questions, also emerged as an important facilitator to overall computer usage.

**Conclusions:**

This pilot study offers important insights into the self-identified barriers and facilitators in computer and Internet use among urban low-SEP novice users as well as the challenges faced by the research team in implementing the intervention.

## Introduction

### Background

The Internet has emerged as a major source of information in the United States. As of 2006, nearly 70% of the American adult population—over 200 million people—reported going online at least on an occasional basis [1]. The Internet is now widely used for communication, shopping, information seeking, and social networking. The Internet has also emerged as a significant source of health information. Almost 80% of Web users have searched for health information on a variety of topics, including diet, fitness drugs, hospitals, new treatments, alternative medicines, and doctors [2-4].

Despite the increasing penetration of the Internet and amount of online health information, there are significant barriers that limit its widespread adoption as a source of health information. One is the “digital divide,” with people of higher socioeconomic position (SEP) demonstrating greater access and usage compared to those from lower SEP groups [5,6]. Online health information seeking is influenced by broadband access and experience in usage [7], and those with less education and income and those who are older are less likely to have broadband connections at home [8]. Broadband access is also influenced by location; urban markets are often serviced by larger companies offering cutting-edge technologies, whereas rural areas are left to rely on smaller companies and are often restricted to slower dial-up services [9].

But even as differences in access are narrowing in urban areas, the plethora of information offered through the Internet and the way it is organized can make its navigation challenging. The number of health-oriented websites runs into the tens of millions. For example, a casual Web search for “cancer” yielded more than 15 million hits [6]. Compounded with the sheer number of hits is the fact that fewer health information websites are designed to cater to the needs of those in the lower SEP groups who are more likely to have lower literacy skills. For example, a recent analysis reported a mismatch between the increasing number of low-literacy users and the number of websites on colorectal cancer that could meet their needs [10,11].

Over time, gaps in broadband access are likely to narrow, especially in urban areas, given the increasing provision of free or discounted wireless or Wi-Fi services to low-SEP neighborhoods, the decreasing cost of technology, and the increasing competition among Internet service providers. As the access gap narrows and more people use the Internet, a shift in research needs to occur to explore other dimensions of communication inequality [12], including how one might improve Internet use skills and website design for a range of audiences. This is particularly important in the case of novice users who may not have the technical skills, experience, or social connections that could help them search for health information using the Internet. The focus of our research is to investigate how members of lower SEP groups, people who currently have limited access, use and experience the Internet.

In an effort to better understand these barriers and explore potential solutions, the authors conducted a feasibility pilot study among 12 low-SEP urban families with no or limited computer or Internet experience. Each family received a free computer system, broadband Internet access, monthly computer training courses, and technical support for 1 year. While the ultimate goal of our project is to understand how to promote health information seeking among low-SEP groups, the purpose of the pilot project was to unearth the challenges to implementing an intervention to promote Internet use among urban low-SEP households. The purpose of this paper is to qualitatively describe participants’ self-identified barriers to computer and Internet use during the 1-year study as well as the challenges encountered by the research team in the delivery of the intervention.

### Structural Influence Model

**Figure 1:**
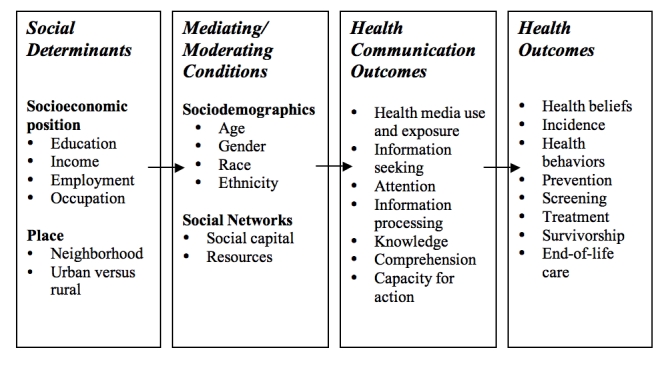
Structural Influence Model [16]

Recent studies have shown that despite the steady improvements in the overall health of Americans, some racial and ethnic minority populations, as well as members of lower SEP groups, experience a lower quality of health services, are less likely to receive routine medical procedures, and have higher rates of morbidity and mortality than non-minorities and those of higher SEP [13]. For example, even as the overall burden of cancer is steadily falling, the decline in both incidence and mortality varies for African Americans, American Indians, and Alaskan Natives compared to Whites [14,15]. Though the connection between health outcomes and social determinants such as social class and race have been well established, the mechanisms connecting them are less clear [12]. The Structural Influence Model starts with the premise that communication is one critical thread that links social determinants with health outcomes and thus provides an overarching theoretical framework for one possible explanation for such disparities in health [16]. We argue that communication plays a central role in promoting preventive behaviors and in influencing patient-provider interactions [12,17-19]. Inequalities in communication among different subgroups may therefore potentially lead to disparate health outcomes among them (Figure 1) [16].

The Structural Influence Model explicitly recognizes the phenomenon that introduction of either new information or new communication technologies may actually have the potential to widen inequalities rather than narrow them. For example, the Knowledge Gap Hypothesis predicts that increasing information flow into a community is more likely to be acquired by high SEP groups at a faster rate compared to lower SEP groups, thus widening gaps in knowledge between them [20,21]. A counterpart to the Knowledge Gap Hypothesis in technology is the digital divide, a well-documented phenomenon where access and use of computers and the Internet is often more prevalent among higher SEP groups than lower SEP groups, thus dividing the world into technology “haves” and “have nots” [8]. Recent surveys on the digital divide report that progress has been made in reducing the gap in Internet access over the last several years in some segments of the US population [22]. However, despite the progress, inequalities still persist in computer and Internet use. Those in the lowest income and education brackets are considerably less likely to be online at home compared to those in higher income and education brackets [8].

Disparities in home Internet access have stimulated efforts to increase public access to computers and the Internet at public libraries and community-based and school-based computer centers [23-25] or through community networks to assist citizens in finding relevant local information [26-28]. Yet, calls are increasingly being made to go beyond simple access to a broader conception of communication inequality, that is, differences in and among social classes in how they access, attend to, process, and use information to improve their health [12]. In the context of the Internet, this implies that research attention should focus on location of access, type of access, and the ability to use the Internet.

While public access has been hailed as a potential solution to the digital divide, home access confers users many important benefits. Individuals with home access report higher levels of empowerment and are more likely to become “active computer users” than those who rely on public access [29,30]. Moreover, people with Internet access at home, especially when they have broadband, spend more time on the Internet and perform more varied online activities [31]. These are key variables to consider when examining such issues as sustainability and dropout rates of overall computer and Internet use over time, which are higher among low-SEP populations [8].

Home Internet access may be particularly critical when issues of privacy and convenience are considered. Individuals may be reticent to communicate online about sensitive conditions when accessing the Internet at public facilities. There are data suggesting that low-SEP populations that have in-home Internet access do use the Internet to seek health information [32,33] and for science-related tasks [34], yet not at comparable rates to those reported by higher-SEP populations [1,34]. As hypothesized by DiMaggio et al (2001), those with higher levels of education and, to a certain extent, higher income possess clear advantages in using the Internet to derive overall occupational, educational, and information benefits [5].

In addition to location, type of access is also important to consider. As cable and telephone companies enter into increasing competition with each other, broadband is slowly replacing dial-up access. The importance of broadband access cannot be overemphasized. Websites are now using sophisticated graphics, interfaces, and software to engage the attention of users. Moreover, in areas such as health, online users can download brochures and information for later use. Last, online users correspond and communicate via patient support groups, blogs, and chat rooms when confronted with health problems or to seek support or even just converse with others with similar conditions. Almost half of broadband users say that their broadband access at home has improved the way that they get health information [2].While enhancing the appeal of the Internet for health, these features also demand faster downloads and broader bandwidth. In fact, availability of broadband, which improves speed and enhances browsing experience, also results in more time spent online compared to experiences with dial-up modems [8].

In short, it is clear that speed, user experience, and location of access, important dimensions of the digital divide, may all enhance user experience and influence Internet use. Despite these important considerations, there have been few empirical investigations of home computer and Internet use, particularly the online health seeking behaviors of novice low-SEP populations. Similarly, there are scant data on the barriers to and facilitators of computer and Internet use among low-SEP populations. Our pilot study explored the feasibility of fielding a study to provide at-home, high-speed Internet access to enhance individuals’ capacity to use the Internet for health-related purposes, while examining the issues that arise from such efforts. An understanding of barriers and issues that are faced by low-SEP groups may allow us to design more effective Internet-based health interventions for the underserved.

## Methods

### Study Design

These data are from the qualitative portion of a pilot pre-post test design trial in which the provision of high-speed Internet access and a computer was the primary manipulation. Urban low-SEP families participating in the pilot study received complete computer systems that included an Apple Mac Mini, a Princeton Series 1510 LCD flat panel monitor, and a Hewlett Packard Deskjet 3740 printer. All computers were equipped with the standard Apple iLife software, which included word processing capability. In addition, typing-practice software was installed on all computers. Participants also received complimentary high-speed cable Internet access for 12 months and completed a mandatory 3-hour introduction computer training class along with optional monthly training classes (approximately 2 hours in length). Participants’ family members were also allowed to attend the trainings. Throughout the study, 24-hour technical assistance was available to all households via a toll-free number; in-home support (provided by a third-party commercial computer support firm) was also available when needed. A member of the research team conducted in-home visits at the start of the intervention to ensure that computers and Internet connections were installed properly.

### Participants

Between August and November 2005, 12 low-SEP urban individuals were recruited through a snowball sampling via advertisements placed in local church bulletins and health care settings, then by referrals from the first group of enrollees. Given our goal of exploring the feasibility of a pilot study, a purposive sample is appropriate at this stage. Individuals were eligible for inclusion if they met the following criteria: (1) no prior home computer or Internet access, (2) limited computer/Internet access outside the home, such as at work or at a public library, (3) at or below 200% federal household poverty line, (4) at least one English-speaking/reading adult age 25-60 years, and (5) at least a fifth grade or above education.

Upon completion of the study, participants were offered the opportunity to complete an in-depth semistructured interview. Each participant was contacted by telephone up to three times to schedule a time for the interview. Nine of the 12 study participants completed the interview process. Three participants did not complete the interview due to scheduling conflicts and lack of available time. The three nonrespondents did not differ on any of the key sociodemographic characteristics or in overall computer and Internet usage compared to the respondents.

### Data Collection

Interviews were approximately 1 hour in length and were conducted by the project director of the study. In exchange for their participation, participants received US $25 in cash upon completion of the interview. The interviews were held in the participants’ homes and were tape recorded for accuracy. Before beginning each discussion, participants verbally gave their informed consent, which was approved by the Human Subjects Institutional Review Board of the Dana-Farber Cancer Institute.

The interviews followed an interview guide that included open-ended questions. The guide was developed by the research team and included standard questions seeking information on participants’ self-identified barriers and facilitators to computer and Internet use both before and during the study. Sample questions are included in Table 2 and Table 3. The semistructured format allowed for the use of probes in which the interviewer could explore participants’ responses to questions in depth as well as delve into research areas of interest that have been established in the literature. For example, there is substantial research citing fear as a reason that people do not engage in computer or Internet use [35]. The interviewer used the probing technique to explore fears that participants may have held prior to the study, as well as the basis for these concerns. The guide also included a series of questions aimed at identifying which aspects of the intervention may have had the greatest role in the overall impact of the study.

The interview guide did not include questions specifically aimed at health information seeking. General tracking data were gathered via Web-tracking software as well as periodic email surveys. The focus of the pilot project was not health information seeking but to explore the feasibility of implementing an intervention study and different ways to gather data on Internet use. The purpose of the in-depth interviews was to gather qualitative information about the barriers and facilitators to the participants’ use of the computer and navigation of the Internet.

Another set of qualitative data was captured to augment the identified challenges and barriers reported by participants in the in-depth interviews. The technical support vendor provided the research team with monthly log reports that detailed each call to the helpline. These logs identified each user/participant, the time and duration of the call, as well as a summary of why the user was placing the call to the tech center.

### Data Analysis

The crystallization/immersion method was used to conduct a qualitative analysis using transcripts from the nine interviews. This method stems from the notion that the researcher is the analytic tool and asserts that vital insights might occur during the data collection process [36]. The crystallization/immersion method is an intuitive analysis style where the researcher organizes data by examining the text thoroughly and then crystallizing out the most important aspects [37]. Two research team members, the principal investigator and the project director, repeatedly read and discussed the transcripts to identify emerging themes and salient topics. Searches for alternative interpretations were conducted and discussed before final decisions were made about how to report and discuss the findings. Once a set of key themes was finalized, links between themes were identified as well as supporting quotations.

## Results

### Lessons Learned

The pilot study offers important insights into the self-identified barriers and facilitators in computer and Internet use among low-SEP novice users as well as the challenges faced by the research team in implementing the in-home intervention.

Nine of the 12 study participants completed the in-depth interview (75% overall response rate), with three men (75% response rate for men) and six women (75% response rate for women) agreeing to be interviewed. Nine of the respondents were black and one was white; 78% (n = 7) of the interview respondents had at least one child under the age of 18 years living in their home for at least half of the study period. The mean household income was approximately US $25000, with an average family size of three (most often including one parent and two children). Eight of the nine interview respondents completed high school and one obtained some college education. Additionally, four of the nine respondents had limited health literacy skills, with Rapid Estimate of Adult Literacy in Medicine (REALM) scores at or below the eighth grade level [38] (Table 1).

**Table 1:** Demographics of interview respondents

Demographic	No. of Respondents (n = 9)
**Age** (years)	
25-60	9
**Gender**	
Male	3
Female	6
**Race**	
African American	8
White	1
**Income** (US $)	
10000-20000	4
20001-30000	3
30001-40000	0
40001-50000	1
50001-60000	1
**Education**	
High school	8
Some college	1
College degree	0
**Baseline Health Literacy (REALM score)**	
3rd grade (0-18)	0
4th-6th grade (19-44)	1
7th-8th grade (45-60)	3
High school (61-66)	5

### Barriers to Internet Use

The main barrier reported by the participants was limited time to allocate to consistent computer use (Table 2). Participants reported that they would have liked to spend more time on the computer and Internet in order to take advantage of all the programs that the computer had to offer. Participants felt that they knew there was potential to “do more” with the computer and the Internet but they felt that they needed more time with it in order to “figure things out.” (see first quote in Table 2).

Interestingly, participants did not report their lack of computer literacy skills such as typing or Web navigation skills as an impediment to their overall computer use, which is often a major barrier cited in the digital divide literature [39]. All participants, however, mentioned that they wanted to continue to improve their computer literacy skills in order to take “full advantage” of all the various features/software the computer and Internet had to offer. A typical response pertaining to this theme is echoed in one participant’s comment:

I wish I could have attended more of the training sessions to learn more skills. I know for a fact that there’s just so much more that I could learn that I do want to learn.

However, participants, both men and women, typically lacked time due to work and the responsibilities of taking care of a family. One female participant who worked at night commented, “I’m sleeping during the day and by the time I get up I don’t have time really to get into the computer.” This theme of limited time is echoed in other digital divide research [28].

Another barrier to computer and Internet use mentioned by all participants was family conflict regarding time spent using the computer (see Table 2). As one participant, a single father, commented,

Time-sharing is always an issue when you have three kids, it’s just something else to manage. But I think we managed.

Conflict was reported between siblings, between parents and children, and between spouses. Typically, the source of conflict was over time with the computer. For example, one grandmother who cares for her two younger grandchildren commented that she never get a chance to use the computer because of the kids (see second quote in Table 2).

And a father mentioned, “The only barrier I have in using the computer is trying to get my kids off of it so I can get on.” Other parents reported that they would rather have their children use the computer for school work since that was seen as more important than using the computer themselves to “surf the Internet.” In general, siblings wanted more time with the computer, parents wanted their children to spend less time doing noneducational activities on the computer, and spouses wanted their counterparts to spend less time with the computer. One of the fathers in the study reported,

My daughter complained that I was on the computer too much. She said ‘You’re always on that. I can never use it.’

Though all of the participants cited some form of conflict, only one participant mentioned that it was a enough of a burden to eliminate the computer from the household.

**Table 2:** Emergent themes for barriers to computer and Internet use

Interview Guide Topic	Emergent Theme	Exemplar Quote
Barriers to computer and Internet use Sample Questions: Have you encountered any barriers in using the computer or Internet? Has anything made it difficult or challenging for you to use the computer or Internet?	Lack of time	“I need to use it more…. I haven’t used it in a while…. I want to learn more on how to use a computer to the fullest because I have a friend who uses Mac and he says things to me where I’m just totally illiterate to it. I’m like, ‘It can do that?’”
	Family conflict	“I never get a chance to use it because of them kids…. I would love to...but my day is kind of busy and I can’t really use it on the weekend…. The kids are on it all the time…and they don’t ever take no for an answer.”

### Facilitators to Internet Use

Participants identified three factors that assisted them in overcoming barriers to their computer and Internet use. Two of the main facilitating factors offered by the participants, training and technical support, were components of the intervention design (see Table 3). The reliance and importance of these variables in overall computer and Internet usage should be considered in the development of future digital divide studies and programs. The other facilitator that was implicitly discussed by study participants was the role of social support that was, in essence, a by-product of the intervention design.

Participants reported that the provision of training courses as well as a technical support service was a central facilitator in helping them to use their computer and the Internet. Participants were somewhat apprehensive at the outset of the study, unsure of their ability to set up the computer by themselves and then properly use it. We required that each participant attend a 3-hour introduction training in which they learned about their computer and how to set it up. According to the participants, this introductory training helped to alleviate these initial concerns and prompted many of them to attend the other optional monthly training courses that the study provided. Participants said that attending the training classes helped them to improve their computer literacy skills such as typing, Web browsing and searching abilities, navigation of their computer programs and applications, and how to use these programs and applications such as word processing software (see first quote in Table 3). And when asked what additional elements participants would have liked to be included in the study/intervention, all those interviewed citied the provision of more training classes. For those not attending all of the sessions, they wished they were able to attend more of the classes. An exemplar quote from one mother in the study pertaining to this theme is

I wasn’t able to attend all of the classes. I’d have liked to have been able to have more classes or more time in classes, like having computer class once a week opposed to once a month [which] may have been more helpful ‘cause everyone in the class would have been able to bring up more ideas, different things, and help one another.

**Figure 2:**
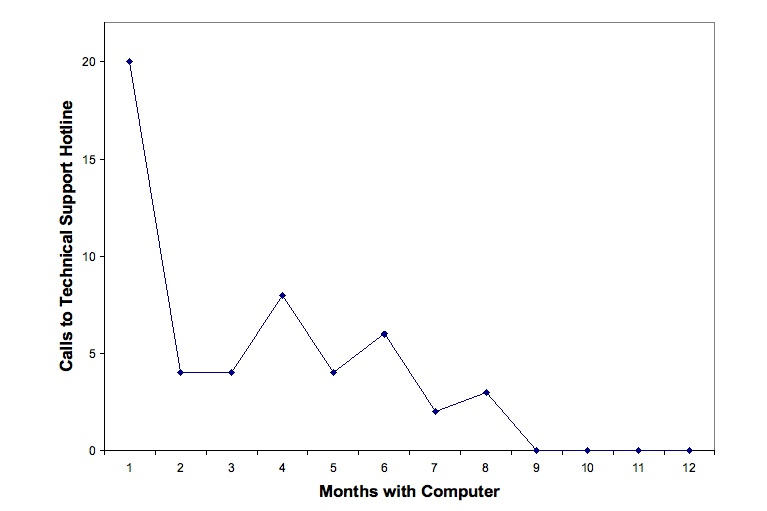
Total number of calls by all participants to technical support helpline by number of months with computer

The other major participant-identified facilitator to computer and Internet use was the availability of a free technical support helpline. The participants were given a toll-free technical support number to call if they encountered any problems or had questions during the course of the study. All participants placed at least one phone call to the helpline during the course of the study, with many of the participants placing at least one phone call per month and some placing as many as 4-7 calls per month. The number of calls to the helpline did lessen over the course of the study, indicating that participants may have felt more comfortable with their computers as the study progressed and that they may have been able to troubleshoot problems by themselves as their familiarity and comfort with the computer increased (Figure 2). This claim is supported by the participants’ own comments when asked about the role of technical support during the in-depth interview. One participant shared,

The first time I called I was clueless, so he remotely figured out the problem. I immediately called [technical support] the first couple of months [but] as I got more comfortable, I could figure it out most of the time on my own and the last couple of times I wrote to Yahoo! directly because I had trouble with their games.

Another reported that he felt more comfortable to upgrade a software after calling technical support (see second quote in Table 3). This concept of gaining confidence over time and learning from prior technical support calls was echoed by almost all of the participants who were interviewed.

Participants also reported that they enjoyed having a reliable source that they could call upon if they had a question or problem. This provided time-sensitive responses to problems and allowed for the continued use of the computer. There was little or no “down time” in which the participants did not have access while they waited for a resolution. This did not mean that the participants solely relied on the technical support vendor for assistance. All of the participants interviewed said that they would first attempt to troubleshoot a problem by themselves. If they could not rectify the issue, the majority of the participants then would ask help from either their older children or spouse. If an issue remained unresolved at this point, they would call the technical support number for assistance. For example, a participant mentioned,

We couldn’t get on the Internet at all. I tried a couple of things then had no idea, and just making a simple phone call [to tech support] helped me to guide me through. Now if something goes wrong I remember what [tech support] said and do it myself. I feel like I am learning.

As illustrated in one of the quotes above, the training sessions not only provided an opportunity for participants to learn computer skills from the instructor but many of the participants reported learning from one another (see third quote in Table 3). Participants stated that they connected with other “students” and subsequently felt better about their novice skill level seeing that they were “not alone.” Similarly, the research team observed participants sharing email addresses with one another and offering advice on how to troubleshoot particular problems they had encountered in the past. This concept of social support and social learning perhaps ameliorating digital divide issues has not been adequately explored in current research and is an aspect that the research team will investigate in future research studies.

**Table 3:** Emergent themes for facilitators to computer and Internet use

Interview Guide Topic	Emergent Theme	Exemplar Quote
Facilitators to computer and Internet use Sample questions: Of the barriers that you have talked about, what would make it easier for you to use the computer or Internet—what would help you overcome these barriers? Has any aspect of the intervention helped to ease any fears you may have had going into the study? If so, what?	Training courses	“The classes made me more confident…. I went home and tried the stuff I learned in the classroom.”
	Technical support	“I was afraid to update ‘cause I’m like ‘What am I updating?’... Now [after calling tech support] I feel more comfortable, you know, as far as downloading things or upgrading any programs that come up.”
	Social support	“Everyone in the class would bring up ideas, different things, and help one another, whatever. One person may have learned it, and the other person didn’t know…you know, just share... share the information with each other.”

### Implementation Lessons

The study offered some important lessons to the research team in implementing a study of this kind. One challenge in implementing an in-home study is the lack of available space in participants’ homes. In most cases, participants lacked enough space to make room for an additional appliance. Moreover, some participants did not have appropriate furniture to hold the computer. After encountering these unanticipated barriers, appropriate furniture (folding table and chair) were provided to those participants in need. Also, a member of the research team assisted with any difficultly in computer setup during the in-home visit at the initiation of the study.

High-speed broadband Internet service was provided to each household via the local cable company. Since all of the participants were already receiving cable services from this company, failure to pay home video or phone cable bills became a logistical problem. If participants were late with their cable bill, the provider terminated their service, which affected their Internet service as well. Placing phone calls to the cable company rectified this issue, but this is a point that warrants attention in any intervention utilizing the Internet as bundled services are becoming more widespread.

## Discussion

Communication is central to learning about health. Inequalities in communication offer one potential explanation for disparities in health among those of diverse sociodemographic backgrounds [6]. One key dimension of communication inequality that affects health outcomes is the divide that exists between those that have access to computers and the Internet and who can properly navigate these resources and those who do not and cannot.

The purpose of the pilot feasibility study was to extend digital divide research beyond the limited idea of access and to examine the barriers and possible facilitating factors that may help to ameliorate disparities in navigation and use of the Internet among an urban low-SEP population. Yet, it is important to note the limitations of the study in considering the implications and applications of the results. Our sample was small and nonrandom, which restricts the generalizability of the findings. The study sample also consisted solely of urban adults and did not explore the persisting urban-rural divide. However, qualitative findings are not designed to be externally valid for population groups at large, but rather consideration of the contextual background provided should allow the reader to ascertain for which situations the findings are most valid [37].

The findings presented in this analysis offer some intriguing lessons for those engaged in promoting adoption of the Internet for health information among urban low-SEP populations.

First, our experiences suggest that additional training and technical support are both critical in enhancing computer usage and navigation of the Internet. In our study, we found that all participants identified training and technical assistance as key supports to their computer and Internet use; particularly in households with children, we suspect that such supports may be necessary to ensure consistent use by adults. Next, novice users gain increasing confidence with limited investment in training and technical support. Even with minimal training, that was optional in nature, we found increasing levels of self-efficacy with respect to navigating the Internet as well as troubleshooting technical problems on one’s own.

We found barriers such as time constraints and family conflicts regarding computer use to be prevalent, though not serious enough to stop individuals from using the computers. However, we found that these conflicts did prevent participants from using the computer as much as they would have liked to if there was no time constraint or family conflict. Future studies should consider providing participants with strategies to manage computer use as well as negotiating techniques to help alleviate any computer-related family conflict.

There are several other key concepts that emerged from the pilot study that should be considered in developing similar research in the future. Social networks and social support emerged as facilitators to solving technical problems and encouraging use. Researchers should examine how computer training sessions may foster the development of new networks and how these networks are utilized in the development of computer literacy for those within the network. Also, living conditions such as space in the home and number of people at home are factors worth taking into account in the design of future studies. Finally, limited economic means is a significant determinant of computer and Internet use. Ability to pay bills for cable, telephone, or even municipal wireless service will influence the continuity of access. Given that these are recurring expenditures, it is extremely important that this be taken into account if all the promises of eHealth are to be fulfilled for the entire population [12].

## Abbreviations

REALM: Rapid Estimate of Adult Literacy in Medicine
SEP: socioeconomic position

## References

[1] Pew Internet & American Life Project February 15 – March 7, 2007 Tracking Survey.

[2] Fox S (2005). Health information online: eight in ten internet users have looked for health information online, with increased interest in diet, fitness, drugs, health insurance, experimental treatments, and particular doctors and hospitals.

[3] Satterlund Melisa J, Mccaul Kevin D, Sandgren Ann K (2003). Information gathering over time by breast cancer patients. J Med Internet Res.

[4] Beckjord Ellen Burke, Finney Rutten Lila J, Squiers Linda, Arora Neeraj K, Volckmann Lindsey, Moser Richard P, Hesse Bradford W (2007). Use of the internet to communicate with health care providers in the United States: estimates from the 2003 and 2005 Health Information National Trends Surveys (HINTS). J Med Internet Res.

[5] DiMaggio P, Hargittai E, Neuman WR, Robinson JP (2001). Social implications of the Internet. Annu Rev Sociol.

[6] Viswanath K (2005). Science and society: the communications revolution and cancer control. Nat Rev Cancer.

[7] Cline R J, Haynes K M (2001). Consumer health information seeking on the Internet: the state of the art. Health Educ Res.

[8] Fox S (2005). Digital divisions.

[9] Inglis J Internet disconnect. The Phoenix. 2007 Aug 22.

[10] Kaphingst Kimberly A, Zanfini Christine J, Emmons Karen M (2006). Accessibility of web sites containing colorectal cancer information to adults with limited literacy (United States). Cancer Causes Control.

[11] Norman Cameron D, Skinner Harvey A (2006). eHealth Literacy: Essential Skills for Consumer Health in a Networked World. J Med Internet Res.

[12] Viswanath K (2006). Public communications and its role in reducing and eliminating health disparities. In: Thomson GE, Mitchell F, Williams MB, editors. Examining the Health Disparities Research Plan of the National Institutes of Health: Unfinished Business.

[13] Agency for Healthcare Research and Quality (2005). 2005 National Healthcare Disparities Report.

[14] Krieger N (2001). Historical roots of social epidemiology: socioeconomic gradients in health and contextual analysis. Int J Epidemiol.

[15] Lynch J (2000). Income inequality and health: expanding the debate. Soc Sci Med.

[16] Viswanath K, Ramanadhan S R, Kontos E Z Mass media and population health: a macrosocial view. In: Galea S, editor. Macrosocial Determinants of Population Health (forthcoming).

[17] Hornik R (2002). Public Health Communication: Evidence for Behavior Change.

[18] Smedley B, Stith A, Nelson A (2003). Unequal Treatment: Confronting Racial and Ethnic Disparities in Health.

[19] Viswanath K, Breen Nancy, Meissner Helen, Moser Richard P, Hesse Bradford, Steele Whitney Randolph, Rakowski William (2006). Cancer knowledge and disparities in the information age. J Health Commun.

[20] Tichenor PJ, Donohue GA, Olien CN (1980). Community Conflict and the Press.

[21] Viswanath K, Finnegan JR (1996). The knowledge gap hypothesis: twenty-five years later. In: Burleson B, editor. Communication Yearbook 19.

[22] Pew Research Center for the People & the Press Americans Going Online ... Explosive Growth, Uncertain Destinations. 1995. http://people-press.org/reports/print.php3?ReportID=136.

[23] Lonergan JM (2000). Internet Access and Content for Urban Schools and Communities.

[24] Roberts L G (2000). Federal programs to increase children's access to educational technology. Future Child.

[25] Breeden L (2001). Connecting Families to Computers and On-Line Networks: A Guide to Key Ideas, Effective Approaches, and Technical Assistance Resources for Making Connections Cities and Site Teams.

[26] Simon ME (1998). Neighborhood Link: A Community Network for Cleveland’s Inner City.

[27] Lentz B, Straubhaar J, LaPastina A, Main S, Taylor J (2000). Structuring Access: The Role of Public Access Centers in the Digital Divide.

[28] Pinkett R (2003). Community technology and community building: early results from the creating community connections project. Inform Soc.

[29] Bishop AP, Tidline TJ, Shoemaker S, Salela P (1999). Public Libraries and networked information services for low-income communities. Libr Inform Sci Res.

[30] Masi Christopher M, Suarez-Balcazar Yolanda, Cassey Margaret Z, Kinney Leah, Piotrowski Z Harry (2003). Internet access and empowerment: a community-based health initiative. J Gen Intern Med.

[31] Tsikalas K, Gross EF (2002). Home computer use among low-income, minority urban adolescents: fulfillment of basic needs and impact on personal and academic development. Paper presented at: Annual Meeting of the American Educational Research Association; April 1-5, 2002; New Orleans, LA; ERIC; 2002.

[32] Kind Terry, Huang Zhihuan J, Farr Deeonna, Pomerantz Karyn L (2005). Internet and computer access and use for health information in an underserved community. Ambul Pediatr.

[33] Wagner Todd H, Bundorf M Kate, Singer Sara J, Baker Laurence C (2005). Free internet access, the digital divide, and health information. Med Care.

[34] Robinson J, DiMaggio P, Hargittai E (2003). New social survey perspectives on the digital divide. IT & Soc.

[35] Fox S (2002). Trust and privacy online: why Americans want to rewrite the rules.

[36] Borkan J (1999). Immersion/crystallization. In: Crabtree B, Miller W, editors. Doing Qualitative Research. 2nd ed.

[37] Malterud K (2001). Qualitative research: standards, challenges, and guidelines. Lancet.

[38] Davis T C, Crouch M A, Long S W, Jackson R H, Bates P, George R B, Bairnsfather L E (1991). Rapid assessment of literacy levels of adult primary care patients. Fam Med.

[39] Lenhart A, Horrigan J, Rainie L, Allen K, Boyce A, Madden M, O’Grady E (2003). The ever shifting Internet population: a new look at Internet access and the digital divide.

